# Varieties

**Published:** 1828-04-01

**Authors:** 


### VARIETIES.

### 1. AMMONIACAL SULPHATE OF COPPER IN EPILEPSY.

In a late Number of Hufeland's Journal, Dr. Urban, of Bernstadt, has published some cases of epilepsy, cured by the above medicine, which, indeed, is not a new remedy. It is to be remembered, however, that Dr. Urban confines this treatment to those who evince no corporeal lesion, as the cause of the disease, and that he puts his patients on a very strict regimen, employing proper evacuations, sanguineous and intestinal, from time to time. These means are, perhaps, not merely auxiliaries, but principals in the methodus medendi.

### 2. MURIATE OF IRON IN SOFTENING OF THE STOMACHS OF CHILDREN.

Dr. Pommer having lost two children affected with vomiting and purging, found, on examination, that the mucous membrane of the stomach was in a state of ramollissement, or softening. He accordingly treated some other infants similarly affected, with the hydrochlorate of iron, and saved them. He was induced to employ this remedy from the recommendation of Professor Autenrieth, who found it very efficacious in arresting the distressing diarrhœas that accompany or supervene on typhus and other bad fevers. We observe, however, that Dr. Pommer has conjoined other means which may have borne a considerable share in the cure. Thus, when the vomiting and purging were severe, he abstracted almost every kind of food and drink, except a few spoonfuls of warm milk twice a day. There being fever present, he applied cold to the head—sinapisms to the legs, and cataplasms to the stomach. After these means, or with them, he employed the hydro-chlorate of iron, and the children recovered. Some cases are detailed in the Heidelberg Annals, which appear to shew the utility of the medicine.

### 3. LIGATURE OF ARTERIES ULTRA TUMORES.

In the ARCHIVES GENERALES for November last, there is given an account of the wonderful success of these operations in this country,—among others Mr. Lambert's case, which is introduced to our Continental brethren in the following terms:—"L'example de WARDROP a été suivi *avec beaucoup de succés*, dans un autre cas d'anevrysme, par le DOCTEUR JAMES LAMBERT." Mr. Lambert's case finishes, in the continental records, most successfully. "La tumeur finit aussi par disparaitre, &c." In the German Journals, all these cases have been published as attended with the most happy results, and German surgeons are called upon to imitate the wonderful exploits of their English brethren! There was a saying, in the olden time, "omnia vincit VERITAS, et prevalebit." We begin to think that the ancients were entirely mistaken, and that the adage should have run thus: "omnia vincit MENDACIUM, et prevalebit." There was a golden age—and there was an iron age—the present appears to be the age of BRASS.

### 4. FATAL HÆMORRHAGE FROM LEECH-BITES.

M. Lisfranc has lately related a case in the Royal Academy of Medicine, which occurred at LA PITIÉ. A female was received into the Hospital with an ulcer, and some symptoms of gastric inflammation. Thirty leeches were applied to the epigastrium, which bled moderately, and then stopped. Three days afterwards the leech-bites opened again, in the middle of the night, and the patient was found dead the next morning, from the profuseness of the hæmorrhage.

